# Diagnosis at gut point: rapid identification of pneumoperitoneum via point-of-care ultrasound

**DOI:** 10.1186/s13089-020-00195-2

**Published:** 2020-12-07

**Authors:** Matthew A. Taylor, Christopher H. Merritt, Philip J. Riddle, Carter J. DeGennaro, Keith R. Barron

**Affiliations:** 1 University of South Carolina School of Medicine, Columbia, SC 29209 USA; 2 Department of Internal Medicine, Prisma Health Midlands, University of South Carolina School of Medicine, Columbia, SC 29209 USA; 3 Department of Emergency Medicine, Prisma Health Midlands, University of South Carolina School of Medicine, Columbia, SC 29209 USA; 4 Ultrasound Institute, University of South Carolina School of Medicine, Columbia, SC 29209 USA; 5 Palmetto Health-USC Medical Group, 5 Medical Park Road, Columbia, SC 29203 USA

**Keywords:** Ultrasound, Pneumoperitoneum, Free air, Intraperitoneal, Colonoscopy, Small bowel perforation

## Abstract

Undifferentiated abdominal pain is a common presentation often requiring immediate medical or surgical intervention. Providing an accurate diagnosis involves a detailed patient history and thorough physical exam. Point of care ultrasound is gaining acceptance as a rapid diagnostic tool that can be used to accurately detect life-threatening conditions while potentially avoiding unnecessary radiation exposure and facilitating rapid treatment. Detection of pneumoperitoneum with point-of-care ultrasound is a simple procedure that relies heavily on the experience of the investigating practitioner. Standard technique involves placing a high-frequency linear-array transducer in the right upper quadrant, where abdominal free air is most likely to accumulate. Detection of the ‘gut point’, which is the transition of abdominal wall sliding to lack thereof in a single image, is the pathognomonic finding of pneumoperitoneum. If visualization is difficult, moving the patient to the left lateral decubitus position or using the scissors technique can provide additional image views. This representative case report and review highlights the use of abdominal POCUS for the diagnosis of pneumoperitoneum. Ultrasound should continue to be explored by clinicians to narrow the differential diagnosis of acute abdominal pain.

## Background

Medical diagnosis, and undifferentiated abdominal pain in particular, is a complex, intricate, and multifactorial process that relies on years of experience and training. Physicians are often challenged to analyze a series of complicated, nonspecific symptoms, and expected to make a diagnostic conclusion. Undifferentiated abdominal pain is a symptom that points to a wide array of pathologies, ranging from benign to life-threatening conditions [1]. A recent study found that 64% of cases with diagnostic errors had undifferentiated chief complaints, such as abdominal pain [2]. Diagnostic errors may be attributed to a myriad of factors including variations in imaging interpretation, patient complexity, and increased case load [3]. These findings highlight the need for expansion of diagnostic methodologies for chief complaints with a broad differential diagnosis.

In patients with severe, acute abdominal pain, the preferred diagnostic tools are primarily multidetector Computed Tomography (MDCT) and abdominal radiography [4]. However, bedside ultrasonography use may be critical for assessment in certain patient populations where CT use should be limited; such as pregnant women, children, and patients with a previous exposure to high radiation [5, 6]. Recent advances in ultrasound technology allow for smaller, faster, and cheaper ultrasound machines to be used as part of the patient encounter at the bedside, termed point-of-care (POCUS) or bedside ultrasound. POCUS is increasingly being used, but investigation into its use in situations of undifferentiated abdominal pain are somewhat limited [7]. These limitations are largely due to the high skillset required to use and interpret ultrasound accurately as a diagnostic tool [8].

One potential etiology of undifferentiated abdominal pain is post-operative pneumoperitoneum, an often life-threatening condition that requires immediate surgical care to abrogate its high risk of mortality [9]. This case report and literature review will discuss the finding of intraperitoneal free air on POCUS led to the diagnosis of perforated small bowel following diagnostic colonoscopy. Then, the procedural technique and diagnostic criteria used to make the diagnosis will be reviewed, discussing how bedside ultrasound can best be used to complement CT and X-ray imaging in making a rapid diagnosis.

## Case presentation

An 81-year-old woman presented to the emergency department with hematochezia. Although she had chronic nonspecific abdominal pain, she denied significant pain on presentation. She had no prior gastrointestinal bleeds, NSAID use, vaginal bleeding, or vaginal discharge. Remaining history was noncontributory. Physical examination was significant for diffuse abdominal tenderness and gross blood on digital rectal exam. Vital signs were unremarkable. Her hemoglobin was 9.9 g/dL, decreased relative to a known baseline of 14 g/dL. The patient was admitted to the hospitalist service for further evaluation of lower gastrointestinal bleeding.

The initial CT of the abdomen and pelvis only showed colonic diverticulosis without any findings of inflammation or free intraperitoneal air. During the first 24 h after admission, the patient required multiple transfusions of packed red blood cells. The gastroenterology team was consulted and ordered nuclear imaging given the large volume blood loss, which revealed no scintigraphic evidence of active GI bleeding. Esophagogastroduodenoscopy (EGD) was subsequently performed and was grossly unremarkable. A colonoscopy was then performed on hospital day 2, noting numerous diverticula, none of which appeared to be bleeding. The plan at that time was to monitor the patient given that she was hemodynamically stable, and her hemoglobin had stabilized without additional evidence of active bleeding.

**Fig. 1:**
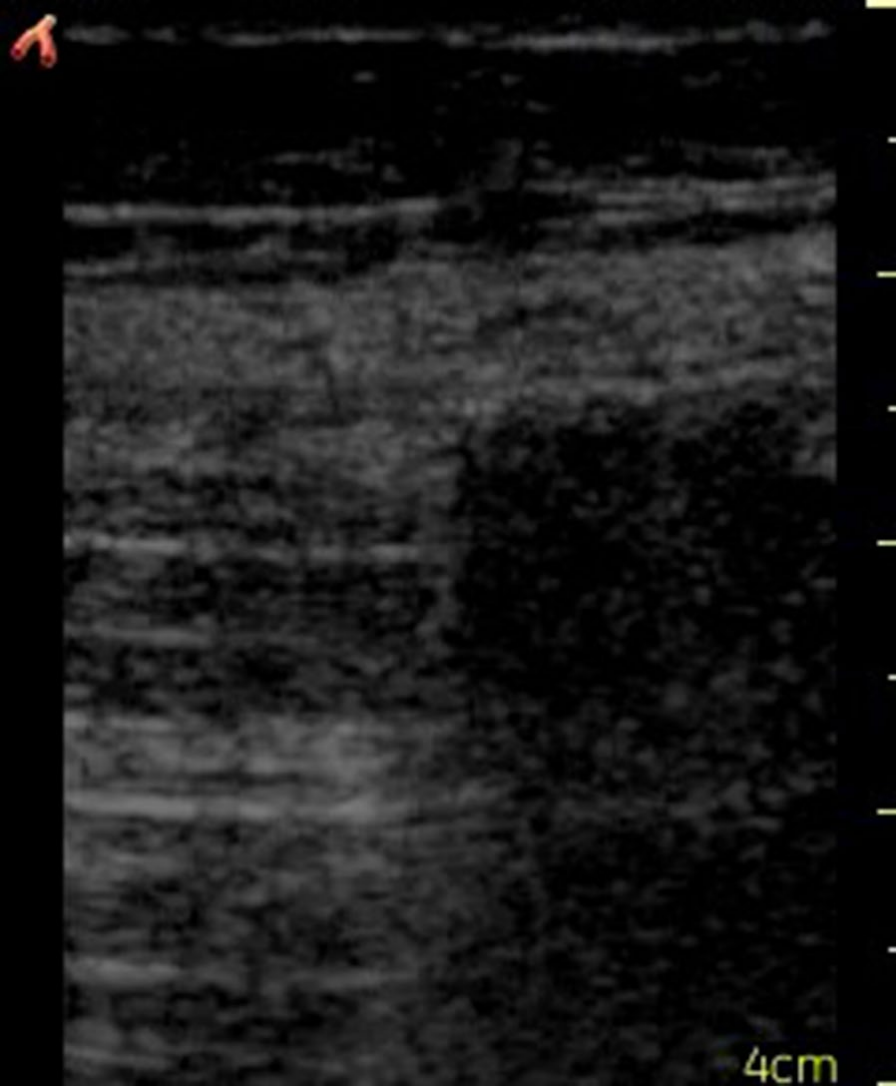
The “gut point” is the transition zone between normal bowel artifact, that may normally contain A-lines, and the abnormal A-line pattern without sliding. Similar to pneumothorax, an absence of sliding with the presence of A-lines is a diagnostic indicator of pathologic free air

**Fig. 2:**
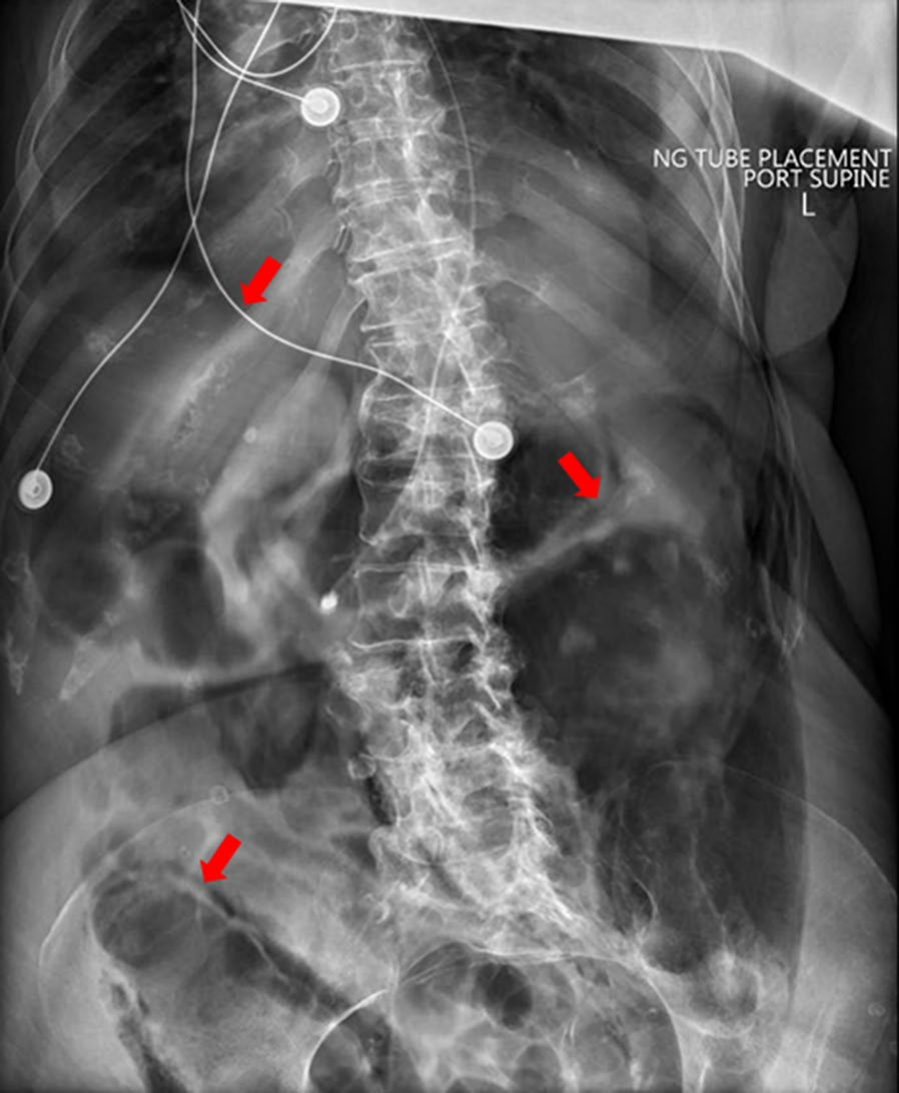
Abdominal X-ray showing free intraperitoneal air due to a small bowl perforation. This image depicts suprahepatic accumulation of free air, which is considered a hallmark sign of pneumoperitoneum

The following day, the rounding hospitalist noted the patient had become acutely confused and complained of worsening abdominal pain. The hospitalist, who has received extensive POCUS training through completion of a primary care ultrasound fellowship [10], performed a bedside ultrasound of the abdomen and immediately noticed a diffuse A-line pattern without sliding of the visceral and parietal peritoneum; a “gut point” indicating a transition zone between free intraperitoneal air and abdominal contents was noted, strongly suggesting pneumoperitoneum (Fig. 1). A general surgeon was consulted, and a subsequent abdominal X-ray was ordered, showing significant free air throughout the abdomen as well as dilated prominent segments of the intestine, suggestive of ileus (Fig. 2). These findings were discussed with family, who wished to forego surgical intervention for probable iatrogenic colonic perforation and elected for conservative management.

Over the next hospital day, the patient had frequent episodes of hypotension and hypothermia and was transferred to the medical ICU, where she was started on vasopressors. She later developed persistent atrial fibrillation with rapid ventricular rate. The palliative care team was consulted, and comfort care was initiated. The patient died on hospital day 6.

## Ultrasonographic identification of pneumoperitoneum

### Technical background

**Fig. 3:**
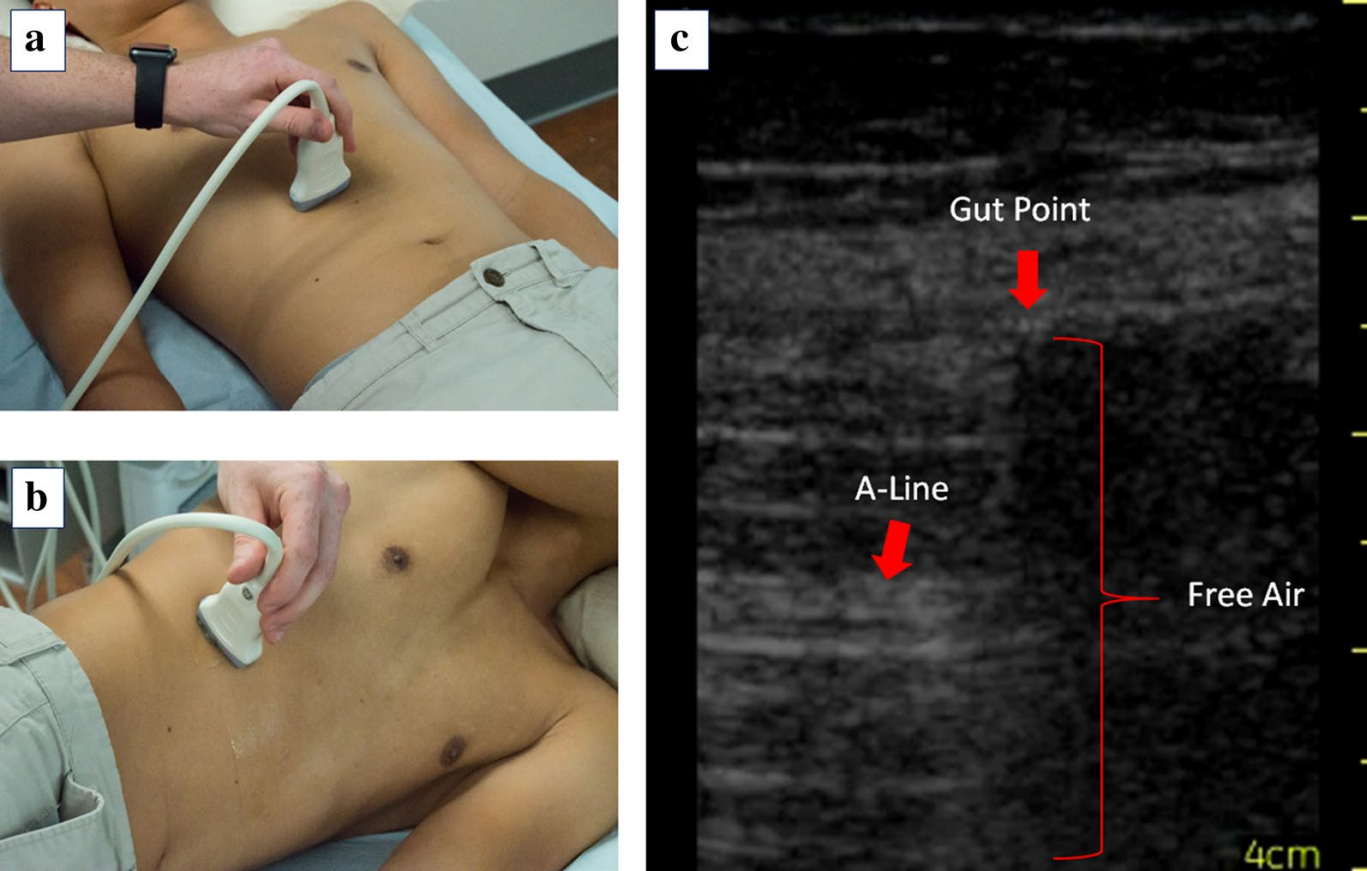
**a** Patient placed in the supine position for an initial sonographic assessment for free intraperitoneal air. **b** Patient placed in the lateral decubitus position to allow free intraperitoneal air to accumulate anterior to the hepatic parenchyma. **c** Sonographic output of a patient with free intraperitoneal air. Air is a strong reflector of ultrasound waves. Reflection produces bright focal lines (A-lines, red arrow) with dark shadowing in between

A tissue–air interface acts as a strong reflector of ultrasound waves. Its strong acoustic reverberations are detected as bright repeating horizontal artifacts. In lung ultrasound, these repeating echogenic artifacts below the pleural line are often termed A-lines [11–13] (Fig. 3).

In the abdomen, tissue–air interfaces are common, given the high air content within bowels in a normal patient, often resulting in multiple horizontal, repeating, echogenic artifacts, particularly over the colon. Thus, physiologic intra-intestinal air may easily be confused with pathologic free abdominal air. To differentiate between the two entities many authors have described different signs, although their varying descriptions may be somewhat confusing to novice ultrasound operators.

For example, intra-intestinal air will be seen moving with peristalsis or respiration. Intraperitoneal free air is generally located under the abdominal fascia and will not be affected by respiration [14]. Abdominal free air produces a strong linear echo with a pathognomonic peritoneal stripe sign [15]. Imaging consists of a single or double echogenic line posterior to the anterior abdominal wall [16]. Of note, visualization may be difficult in obese patients [17].

### Standard technique

Patients should be placed in the supine position to allow free air to rise just beneath the anterior abdominal wall. Either a convex or linear transducer can be used, but the high-frequency (4–8 MHz) linear transducer is preferred. This will produce a shallow, but high-resolution image of anterior abdominal gas pockets while reducing interference of deep intestinal air [18]. Right upper quadrant imaging will provide the highest chance of detection, because free air will most likely accumulate anterior to the liver (Fig. 3a). It may be difficult to differentiate between intestinal air and abdominal free air due to similar appearing reverberation artifacts. If this is the case, the patient can be placed in the left lateral decubitus position for at least 2 min (Fig. 3b), encouraging free air to move to the area of least resistance and causing a shift of the reverberation artifacts. Any potential free air can be more easily seen around the hepatic parenchyma as hyperechoic reverberations that fluctuate with abdominal compression [19].

### Abdominal sliding and “gut point”

For ultrasound operators familiar with lung ultrasound, a “gut point” may be thought of as analogous to a “lung point” found in pneumothorax [20]. In normal patients, a subtle sliding or shimmering is present along the peritoneal line, indicating apposition of the visceral and parietal peritoneum. The pathologic presence of intrabdominal air separates these structures, abolishing this artifact if free air abuts the peritoneum (Fig. 1) [11]. The presence of sliding allows the examiner to differentiate between pathologic abdominal free air and physiologic bowel gas, both of which generate repeating horizontal artifacts similar to A-lines. Sliding the probe laterally, a “gut point” may be seen at the transition point where both abdominal sliding and the absence thereof can be seen in a single ultrasound image (Figs. 1, 3c). Obtaining this image is a diagnostic hallmark of pneumoperitoneum [11] (Additional file 1: Video S1).

### Scissors maneuver

The scissors maneuver can also be used to help detect intraperitoneal free air. It involves lightly placing a linear-array transducer parasagitally in the right epigastric region with the patient lying supine. The transducer should be lightly placed on the abdomen with careful consideration to not compress the skin surface. If reverberation artifacts are seen with a suspicion of abdominal free air, gently press the caudal end of the probe onto the abdomen. This should press the free air away from the anterior liver. The reverberation artifacts that were previously obstructing the liver should now be less prominent. Releasing the pressure from the probe should allow the free gas to return and make the reverberation artifacts more visible [21].

## Discussion

Pneumoperitoneum arises from a variety of causes in both operative and non-operative settings. Exact pathophysiology varies, but generally pneumoperitoneum is caused by a perforated hollow viscus in 85–90% of cases [22]. Recently, pneumoperitoneum has seen an increased incidence due to the greater utilization of minimally invasive endoscopies; leading to a bowel perforation in approximately 1.5 out every 1000 procedures (0.15%) [23]. The greatest independent risk factors include patients over 75 years old and those undergoing therapeutic colonoscopy [24, 25]. The differential diagnosis of non-operative causes of pneumoperitoneum is vast, including Crohn disease, diverticulitis, peptic ulcer disease, and malignancy [26–29]. Peptic ulcer disease and diverticulitis are the leading causes of gastrointestinal perforation (16%), followed by trauma (14%), malignancy (14%), and endoscopy (4%) [30]. Positive clinical outcomes are highly dependent on rapid identification and subsequent surgical intervention [31].

Most cases of suspected pneumoperitoneum are evaluated with CT or radiography [4]. CT is the gold standard for diagnosis but is often preceded by long wait times and unnecessary radiation exposure. A study by Chen and colleagues of 188 patients demonstrated that POCUS for identifying pneumoperitoneum can provide better sensitivity and diagnostic accuracy than abdominal radiography. Overall, ultrasound had improved sensitivity over radiography (92% vs. 78%) but similar specificity (both 53%). They found that both radiography and ultrasound had detection rates approaching 100% when a large amount of abdominal free air was present, but for detecting small amounts of free air originating from pathologic micro-perforations, ultrasound surpassed radiography in accuracy [12]. This can be attributed to the bright echogenic appearance of air on ultrasound that is detectable with air volumes as little as 1 mL [32].

The major downfall of POCUS is its heavy reliance on the technical skills of the operator. A prospective observational study asked four senior physicians with ultrasound experience and two internal medicine residents with no ultrasound experience to blindly interpret both ultrasound and radiographic images of patients with and without pneumoperitoneum. The study concluded that ultrasound had a higher sensitivity for detecting pneumoperitoneum compared to radiography (95.5% vs. 72.2%), but had a lower specificity (81.8% vs. 92.5%) [8]. These results are similar in comparison to previous studies concluding ultrasound surpasses radiography in sensitivity but does not have an advantage in specificity [12]. The diagnostic accuracy of internal medicine residents varied compared to senior physicians with ultrasound experience. Even though both cohorts did not have specific training in diagnosing pneumoperitoneum, senior physicians had a diagnostic accuracy of 89% compared to 68% accuracy by the residents. The authors emphasize that minimal ultrasound training can greatly improve a physician’s diagnostic efficacy.

Many patients with risk factors for pneumoperitoneum may fall under medical, rather than surgical, care. This highlights the importance of not limiting pneumoperitoneum as a diagnostic consideration to surgical floors and emergency departments. Hospitalists and intensivists should consider pneumoperitoneum in patients presenting with undifferentiated abdominal pain with an additional history of abdominal pathology. To account for its low prevalence, ultrasound can be used as a rapid diagnostic tool to differentiate life-threatening pneumoperitoneum while also preventing unnecessary radiation exposure [5]. Such use of ultrasound may be particularly valuable in resource-poor settings, where access to more-advanced diagnostic imaging modalities may be limited.

## Conclusion

Undifferentiated abdominal pain is a common diagnostic dilemma that may not always present with a clear answer. Understanding the basic technique of the abdominal ultrasound exam, along with the ability to differentiate between intestinal and free abdominal air provides clinicians with an additional imaging modality to rapidly detect life-threatening cases of acute pneumoperitoneum. Due to its high sensitivity, low cost, and safety profile, abdominal ultrasound should be considered a first-line imaging modality for diagnosing pneumoperitoneum, as demonstrated in the previously described case presentation. While already widely embraced by emergency medicine, improved ultrasound education in the primary care and surgical specialties will enable the confidence and proficiency to fully realize the scope of ultrasound as a powerful diagnostic tool.

## Supplementary information

**Additional file 1: Video S1.** The “gut point” is the transition zone between normal bowel artifact, that may normally contain A-lines, and the abnormal A-line pattern without sliding. Similar to pneumothorax, an absence of sliding with the presence of A-lines is a diagnostic indicator of pathologic free air.

## Data Availability

Data sharing is not applicable to this article as no datasets were generated or analyzed during the current study.
